# Development of a split-luciferase assay to establish optimal protein secretion conditions for protein production by Bacillus subtilis

**DOI:** 10.1099/mic.0.001460

**Published:** 2024-06-07

**Authors:** Mariah B.M.J. Kes, Biwen Wang, Peter van Ulsen, Leendert W. Hamoen, Joen Luirink

**Affiliations:** 1 Molecular Microbiology, Amsterdam Institute of Molecular and Life Sciences, Vrije Universiteit Amsterdam, De Boelelaan 1108, 1081 HZ, Amsterdam, The Netherlands; 2 Bacterial Cell Biology, Swammerdam Institute for Life Sciences, University of Amsterdam, Science Park 904, 1098 XH, Amsterdam, The Netherlands

**Keywords:** *Bacillus subtilis*, heterologous protein production, NanoBiT, protein detection toolbox, protein secretion, split-luciferase assay

## Abstract

*Bacillus subtilis* is a Gram-positive bacterium that is frequently used in the bioindustry for the production of various proteins, because of its superior protein secretion capacities. To determine optimal conditions for protein secretion by *B. subtilis*, a quick and sensitive method for measuring protein secretion is crucial. A fast and universal assay is most useful for detecting diverse proteins in a high-throughput manner. In this study, we introduce a split-luciferase-based method for measuring protein secretion by *B. subtilis*. The NanoBiT system was used to monitor secretion of four different proteins: xylanase A, amylase M, protein glutaminase A, and GFP nanobody. Our findings underscore the split-luciferase system as a quick, sensitive, and user-friendly method.

**Impact Statement**

The bioindustry plays a crucial role in supplying a wide array of proteins utilized in various applications. Among the key producers of these proteins is the Gram-positive bacterium *Bacillus subtilis*. Accurate detection of the secreted proteins is required to analyse the effect of interventions to enhance protein production. In this study, we introduce the split-luciferase assay for rapid and highly sensitive detection of secreted proteins, irrespective of the protein of interest. This innovation represents a valuable addition to the protein detection toolbox.

## Introduction

The Gram-positive bacterium *Bacillus subtilis* is commonly used for industrial protein production [1]. It possesses a high intrinsic secretion capacity, facilitated by its single membrane, and many of its products have a GRAS (generally recognized as safe) status. Although *B. subtilis* produces a variety of proteins in the bioindustry, the production of heterologous proteins has resulted in variable yields [2]. Therefore, numerous studies have focused on enhancing the yields through the use of various expression hosts, gene constructs, and cultivation conditions [3,4]. These strategies can be successful for a certain protein of choice, but often require individual tweaking of the available biotechnological tools for optimal production.

To identify favourable conditions that enhance protein production and translocation across the cytoplasmic membrane, it is essential to assess protein levels in both the cell and medium fractions using assays that are fast, generic, and robust. Conventional protein detection methods, such as SDS-PAGE, Western blotting, and enzyme activity assays [5], involve labour-intensive protocols, and require either specific antibodies that may not be readily available or enzymatic activity of the studied protein that can be easily monitored. These constraints may delay the analysis of diverse conditions and mutants. Furthermore, these assays often test conditions on a small scale and are difficult to scale up to a high-throughput format.

To circumvent these limitations, an alternative approach is to fuse the secreted protein with a fluorescent protein, such as GFP, and assess protein production by quantifying fluorescence. However, the bulky nature of GFP influences protein secretion and the fluorescently active structure requires time to fold properly: up to 53 min for wild-type GFP [6]. Split versions of the fluorescent protein are available to abate the influence on protein secretion [7,8]. In this technique, the fluorescent protein is split into two non-fluorescent parts: a small peptide, which is fused to the protein of interest and less likely to hamper secretion, and the remainder of the GFP protein, which is added to the samples after secretion of the fusion protein. The small peptide and remaining GFP will then reconstitute into a functional fluorescent protein and report on the amount of secreted fusion protein. However, the reconstitution of GFP and stabilization of the maximal signal requires 16–72 h of incubation time [7, 9,10]. Additionally, the sensitivity of this reporter is not adequate for the detection of low protein levels [11,13].

In contrast to fluorescence-based methods, bioluminescence is a rapid reaction that generates light as a result of a chemical reaction by the enzyme luciferase [14]. This protein produces an excited state of its substrate using oxygen, after which the product releases energy in the form of light to return to the ground state. Luciferase can serve as a reporter for protein production and secretion, offering several advantages over fluorescent reporters: reduced background in standard assay conditions and therefore a higher signal-to-noise ratio and sensitivity, broad linearity of the detection range, and short maturation time [15,16]. The output is also compatible with high-throughput analyses. When luciferase is used as a tag for monitoring protein secretion, a split version can be used to mitigate the potential obstruction of secretion by the bulky luciferase, in analogy to the split-GFP assay described above.

In this study, we employed a split-luciferase assay to measure protein secretion in *B. subtilis*. Specifically, we utilized the split version of NanoLuc luciferase [17], called NanoBiT [18,19]. NanoLuc is an optimized luciferase that is small in size (19 kDa) and produces bright, stable luminescence (half-life >2 h). The split version comprises a small peptide (11 amino acids) called HiBiT and the remaining luciferase protein (18 kDa) called LgBiT. When combined, they reconstitute with high affinity (*K*_D_=700 pM) into the native, active NanoLuc luciferase, and the activity can be measured within minutes. The HiBiT peptide can be fused to a protein of interest to conveniently study its production.

The split-luciferase system has previously been employed to investigate bacterial protein secretion, including *in vitro* studies on Sec-mediated secretion [20], and *in vivo* to investigate type III secretion in *Salmonella* [16]. In this study, our aim was to adapt the assay for *B. subtilis* to provide a rapid and sensitive method to monitor protein secretion in this biotechnologically relevant organism. Additionally, we scaled the assay to a medium-throughput format, enabling the analysis of protein secretion under various conditions over time. This assay can be utilized to compare different settings that may influence secretion efficiency, such as host mutants, signal sequences, growth conditions, and compounds added to the growth medium, and may be extended to other production organisms.

## Methods

### Strains, plasmids, and growth conditions

*B. subtilis* BWB143 (wild-type BSB1, trp+, Δ*aprE*, Δ*nprE*, Δ*spoIIE*) was used as the host strain in the expression experiments. The cells were grown in lysogeny broth (LB) medium (1 % (w/v) NaCl, 1 % (w/v) tryptone, 0.5 % (w/v) yeast extract) supplemented with 25–50 µg ml^−1^ kanamycin to maintain the plasmids, and 0.5 % (w/v) glucose was added to the medium when precultures were grown. Up to 1 % (w/v) of xylose was added to the cultures to induce expression of the constructs. The list of plasmids used in this study is shown in Table 1. *E. coli* strain Top10F′ was used for molecular cloning and was grown in LB supplemented with 100 µg ml^−1^ ampicillin to maintain the plasmids. Chemically competent *E. coli* cells [21] and naturally competent *B. subtilis* cells [22] were used for plasmid transformations.

**Table 1.** Plasmids used in this study

Plasmids	Promotor	Gene
pMKX01	P_xylR_	Empty vector
pDEXH01	P_xylR_	XynAss-XynA-HiBiT
pDEXH02	P_xylR_	AmyMss-AmyM-HiBiT
pDEXH03	P_xylR_	YoaWss-GFPnb-HiBiT
pDEXH04	P_xylR_	YoaWss-PrgA-HiBiT
pMKX09	P_xylR_	Pelss-AmyM-HiBiT

For detailed information on strain and plasmid construction, see Methods S1. All constructs were first transformed to *E. coli* Top10F′ for validation of the constructs by sequencing (EZ-Seq; Macrogen Europe) and subsequently transformed to *B. subtilis* BWB143.

Cells were grown either in Erlenmeyer flasks in a water bath at 37 °C 210 r.p.m, in screw cap tubes (30 ml, Starstedt) in a dry incubator at 37 °C/200 r.p.m or in a 96-well round bottom microtitre plate in a plate reader (BioTek Synergy HTX) at 37 °C with continuous linear shaking.

### Small-scale expression experiments

Bacterial strains were grown overnight in a dry shaker at 37 °C/200 r.p.m. The precultures were diluted to an equivalent of 0.05 OD_600_ units in prewarmed LB and grown in the water bath. After 3 h, xylose was added to 1 % (w/v) to induce the expression of the HiBiT-tagged constructs. Culture and/or supernatant samples were taken at desired time points.

### Medium-throughput secretion monitoring

Bacterial strains were grown overnight in a dry shaker at 37 °C/200 r.p.m. The next day, the cells were diluted 20-fold in 2 ml fresh LB, grown for 2 h at 37 °C/200 r.p.m, and then diluted to an equivalent of 0.05 OD_600_ units and distributed across a 96-well plate (150 µl/well). The cells were incubated in a plate reader at 37 °C with continuous shaking. Every 30 min the OD_600_ was measured. After 2 h, induction of gene expression was induced by addition of xylose to the cultures. At appropriate time points, 5 µl culture samples were taken, diluted in LB supplemented with phenylmethylsulfonyl fluoride (PMSF; 10 mM), and stored on ice until the luminescence was determined. To obtain sufficient samples for protein analysis by SDS-PAGE and Western blotting, the contents of multiple wells were pooled.

### Luminescence detection

The diluted culture samples that were kept on ice, containing secreted, HiBiT-tagged proteins, were mixed with 5 µl of the Nano-Glo HiBiT Extracellular Detection System (Promega) in a black, clear bottom 384 wells plate (Greiner Bio-One) and incubated for 10 min. The luminescence signal was recorded in a plate reader (BioTek Synergy H1), optics type ‘luminescence fibre’, 1 s integration time.

### SDS-PAGE and (far-)Western blotting

Medium fractions were obtained by spinning culture samples for 1 min at 21 300 RCF. For the precipitation of secreted proteins, the supernatant was mixed with an equal volume of 20 % TCA/50 % acetone and incubated for minimally 5 min on ice (maximally overnight, at 4 °C).

The samples were then centrifuged for 20 min at 21 300 RCF at 4 °C. Pellets were washed with 500 µl ice-cold 100 % acetone, dried at RT for 5 min, resuspended in 2× sample buffer (0.125 M Tris pH 6.8, 4 % SDS, 20 % glycerol, 0.02 % bromo phenol blue, 20 uM DTT), 1 : 1 diluted in PBS and heated at 98 °C for 10 min in a heat block.

Protein samples were separated by SDS-PAGE and the proteins were transferred to a nitrocellulose membrane. For far-Western blotting, the nitrocellulose membrane was incubated for 15 min in TBS-T and then 1.2 ml Nano-Glo reagent was added to the membrane and incubated for 5 min. The luciferase signal was quantified in an AI600 imager with 30 s imaging time. For immunoblotting, the nitrocellulose membrane was incubated with blocking buffer [1 % (w/v) skim milk powder in TBS-Tween20] for 1 h at RT or overnight at 4 °C, subsequently incubated with α-HiBiT (1 : 10,000, Mouse, Promega) for 1.5 h or α-AmyM (1 : 10,000, Rabbit, [23]) for 1 h, washed three times with blocking buffer for 10 min in total, incubated with HRP-tagged secondary antibody [goat-anti-mouse (1 : 5,000, Bio-Rad) or goat-anti-rabbit (1 : 10,000, Rockland)] for 1 h, washed three times with TBS-T for 10 min in total, washed with demi-water, and incubated with Lumi-Light Western blotting substrate (Roche) for 1 min followed by chemiluminescence quantification in the AI600 imager. The signal intensities were quantified using Image Lab Software (Bio-Rad).

## Results and discussion

### Development of a medium-throughput split-luciferase assay

To assess the applicability of the split-luciferase system to quantify proteins secreted by *B. subtilis*, we first tagged the native, well-secreted protein xylanase A (XynA) [24] at its C-terminus with the HiBiT peptide. We expressed the xylose-inducible construct in the *B. subtilis* strain BWB143 (wild-type BSB1, trp+, Δ*aprE*, Δ*nprE*, Δ*spoIIE*). This strain has a sporulation-negative phenotype and is deficient in the two major extracellular proteases. These characteristics are advantageous for industrial fermentations. The tagging of XynA with the HiBiT peptide did not alter the expression and secretion of XynA as judged by Coomassie brilliant blue (CBB)-stained SDS-PAGE (Fig. S1a, available in the online version of this article). To evaluate the detection of the luminescence signal, we combined culture samples containing secreted XynA-HiBiT taken at early stationary phase with the Nano-Glo HiBiT Extracellular Detection System (Promega), which contains LgBiT and the NanoLuc substrate furimazine. The reconstitution of LgBiT and HiBiT results in an active NanoLuc luciferase, which catalytically converts furimazine into furimadine, resulting in luminescence. The reconstitution can only occur for secreted XynA-HiBiT, since LgBiT cannot cross the membrane. Following a 10 min incubation period, the luminescence signal was clearly detectable, with a neglectable background signal observed in the culture samples of empty vector control cells (Fig. S1b). Thus, the split-luciferase assay appears effective in rapidly identifying secreted XynA by *B. subtilis*.

High concentrations of the secreted HiBiT-tagged XynA rapidly exhausted the LgBiT and/or substrate furimazine (reagent components) in the Nano-Glo reagent. We therefore tested whether dilutions of samples would lead to a quantitative output. For instance, our results showed that we needed to dilute fully induced XynA-HiBiT samples at least fivefold to obtain a signal within the linear detection range (Fig. S1b). This underscores the assay’s sensitivity.

**Fig. 1.**
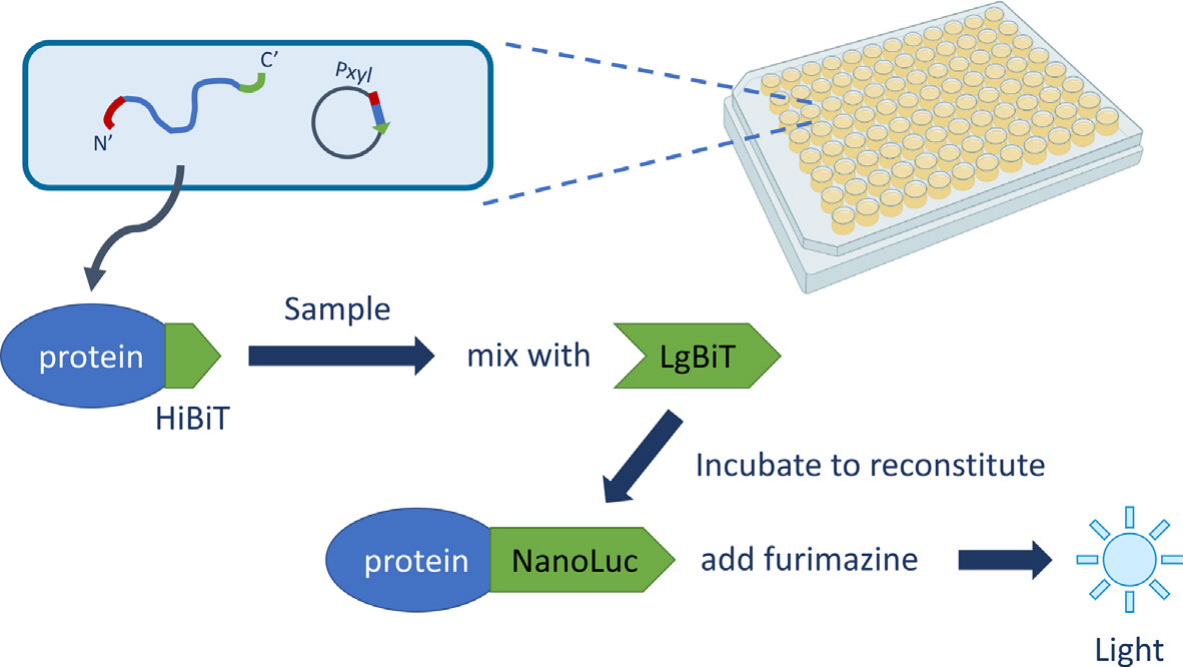
Overview of medium-throughput secretion protocol using split luciferase. Cells harbouring a plasmid encoding the HiBiT-tagged construct are grown in a 96-well plate. The expression of the construct is induced by the addition of xylose and the cells will produce and secrete the HiBiT-tagged protein of interest into the growth medium. At specific time points, samples are taken, diluted in LB, and left on ice until incubation with Nano-Glo reagent, to enable bioluminescence detection using a plate reader. Figure adapted from [34].

To facilitate the application of this assay in screening multiple conditions, strains, and constructs, we established a protocol for monitoring protein secretion in a medium-throughput format (Fig. 1). In this set-up, cells harbouring the expression construct of a protein of interest with the HiBiT tag were first grown in a batch culture for 2 h, followed by dilution to an equivalent of 0.05 OD_600_ units in a 96-well plate for growth in a plate reader until the end log phase was reached. At that point, expression of the construct was induced, in our case with xylose to maximally 1 % (w/v), and culture samples were taken from the wells at designated time points for luminescence measurements using a plate reader.

**Fig. 2.**
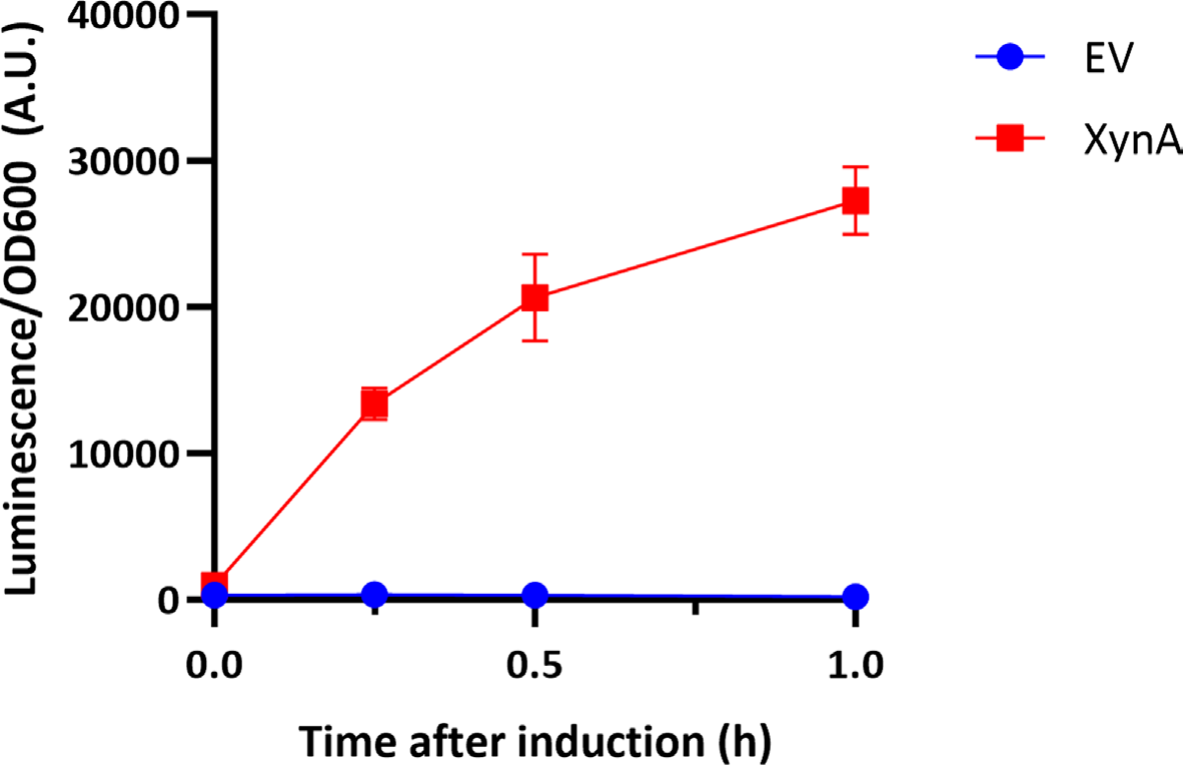
Luminescence signal of secreted XynA-HiBiT. BWB143 cells harbouring either pMKX01 empty vector (EV) or pDEXH01 XynA-HiBiT were cultured with 1 % (w/v) xylose to induce gene expression. Samples were taken at indicated time points and diluted 10-fold in LB and mixed with Nano-Glo reagent. After 10 min incubation time the luminescence signal was measured in a plate reader. Error bars depict standard deviation of three biological replicates for XynA-HiBiT and two biological replicates for the negative control.

In our first medium-throughput experiment, we tested the secretion of XynA-HiBiT and sampled the culture at various time points after induction with 1 % (w/v) xylose (Fig. S2). The secreted protein was detected by bioluminescence as early as 15 min after induction and the signal increased over time (Fig. 2). During the experiment a neglectable background signal was detected in samples from the strain harbouring the empty vector. Of note, a high-throughput setup, in which cells are grown in medium supplemented with the Nano-Glo reagent for continuous luminescence measurement during protein secretion, proved impractical due to rapid depletion of the reagents, although this set-up may work for poorly secreted proteins.

### Comparison of secretion of four distinct proteins

We validated the medium-throughput format by assessing the secretion of four distinct proteins of biotechnological interest that were C-terminally tagged with HiBiT. We chose proteins that vary in host origin, size, and possibly kinetics of secretion by *B. subtilis*. These differences should be detectable by the split-luciferase system to serve its purpose as a screening platform. Besides XynA (20.4 kDa, *B. subtilis*), we tested the heterologous proteins protein glutaminase A (PrgA, 32.7 kDa, *Chryseobacterium proteolyticum* [25]), a nanobody targeting GFP (GFPnb, 13.7 kDa, *Lama glama* [26]), and amylase M (AmyM, 75.2 kDa, *Geobacillus stearothermophilus* [27]). AmyM and XynA were fused to their respective native signal sequence. PrgA and GFPnb were fused to the YoaW signal sequence that is native to *B. subtilis*, and which has previously been shown to efficiently mediate the secretion of the heterologous proteins PhoA, GFP-specific single-chain variable fragment antibody, and RNase barnase [28].

**Fig. 3.**
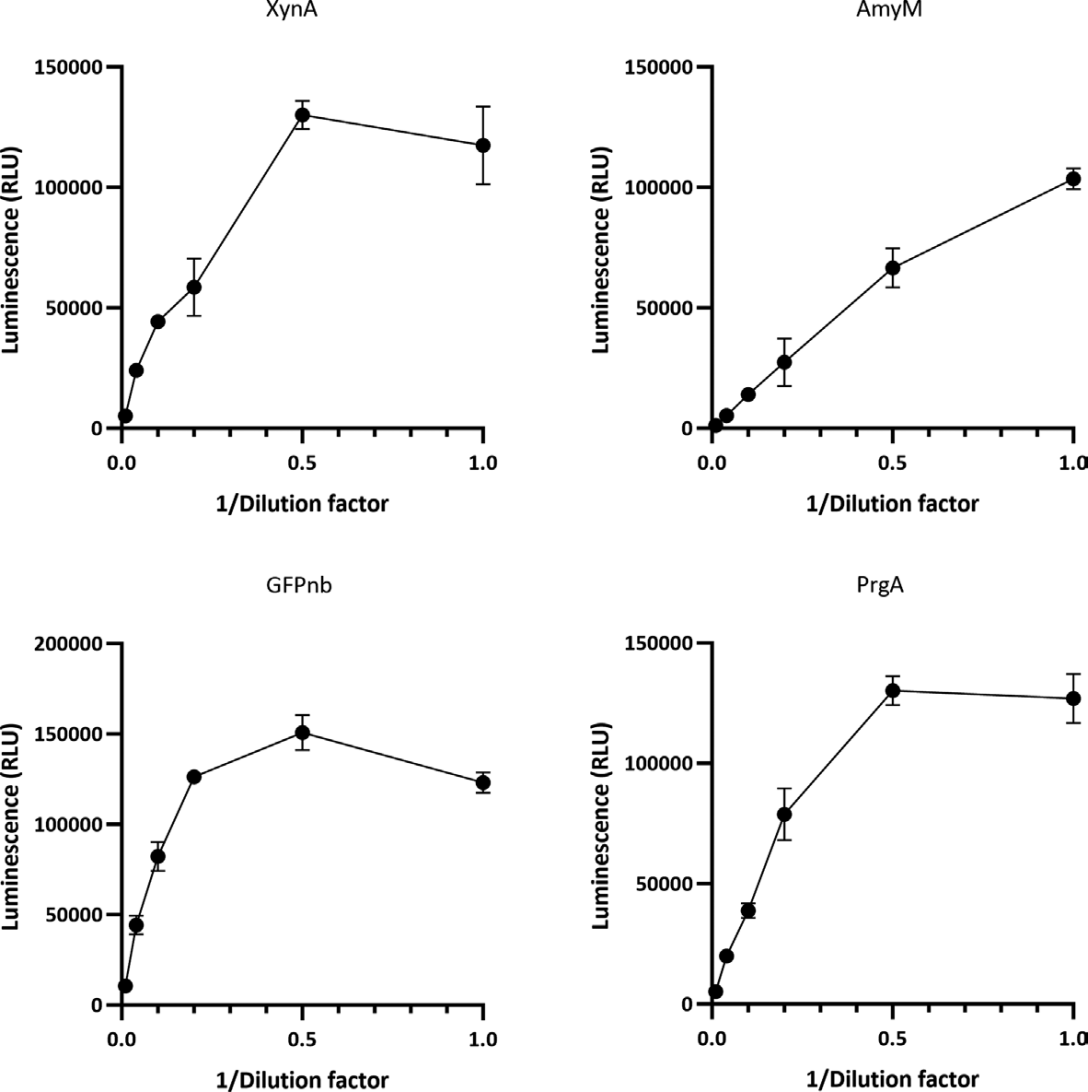
Effect of the dilution of XynA-HiBiT, AmyM-HiBiT, GFPnb-HiBiT, and PrgA-HiBiT samples prior to luminescence measurements. BWB143 cells harbouring either pDEXH01 XynA-HiBiT, pDEXH02 AmyM-HiBiT, pDEXH03 GFPnb-HiBiT or pDEXH04 PrgA-HiBiT were grown from an equivalent of 0.05 OD_600_ units to end log phase, after which expression of the constructs was induced with 1 % (w/v) xylose. Culture samples were taken 4 h after induction and diluted in LB. The diluted samples were incubated with Nano-Glo reagent for 10 min and luminescence was measured in a plate reader. Error bars depict standard deviation of two technical replicates.

Prior to analysing the production dynamics of these four proteins, the appropriate dilution of each sample was determined, to prevent quick exhaustion of the reagent components. *B. subtilis* strain BWB143 carrying the respective plasmids were grown in 10 ml LB in a water bath at 37 °C with shaking at 210 r.p.m. At the end of the log phase, xylose was added to a final concentration of 1 % (w/v) to induce the expression of the constructs. Culture samples were collected 4 h post-induction and serially diluted, and bioluminescence signals were measured in a plate reader (Fig. 3). At this time point, XynA and PrgA samples appeared to require at least a twofold dilution, whereas the GFPnb sample required a fivefold dilution. AmyM, being the least secreted protein, exhibited a signal roughly within the linear range without requiring sample dilution. To facilitate the detection of potentially higher protein levels in follow-up experiments, the standard dilution of each sample in the medium-throughput experiment was set at 10-fold.

**Fig. 4.**
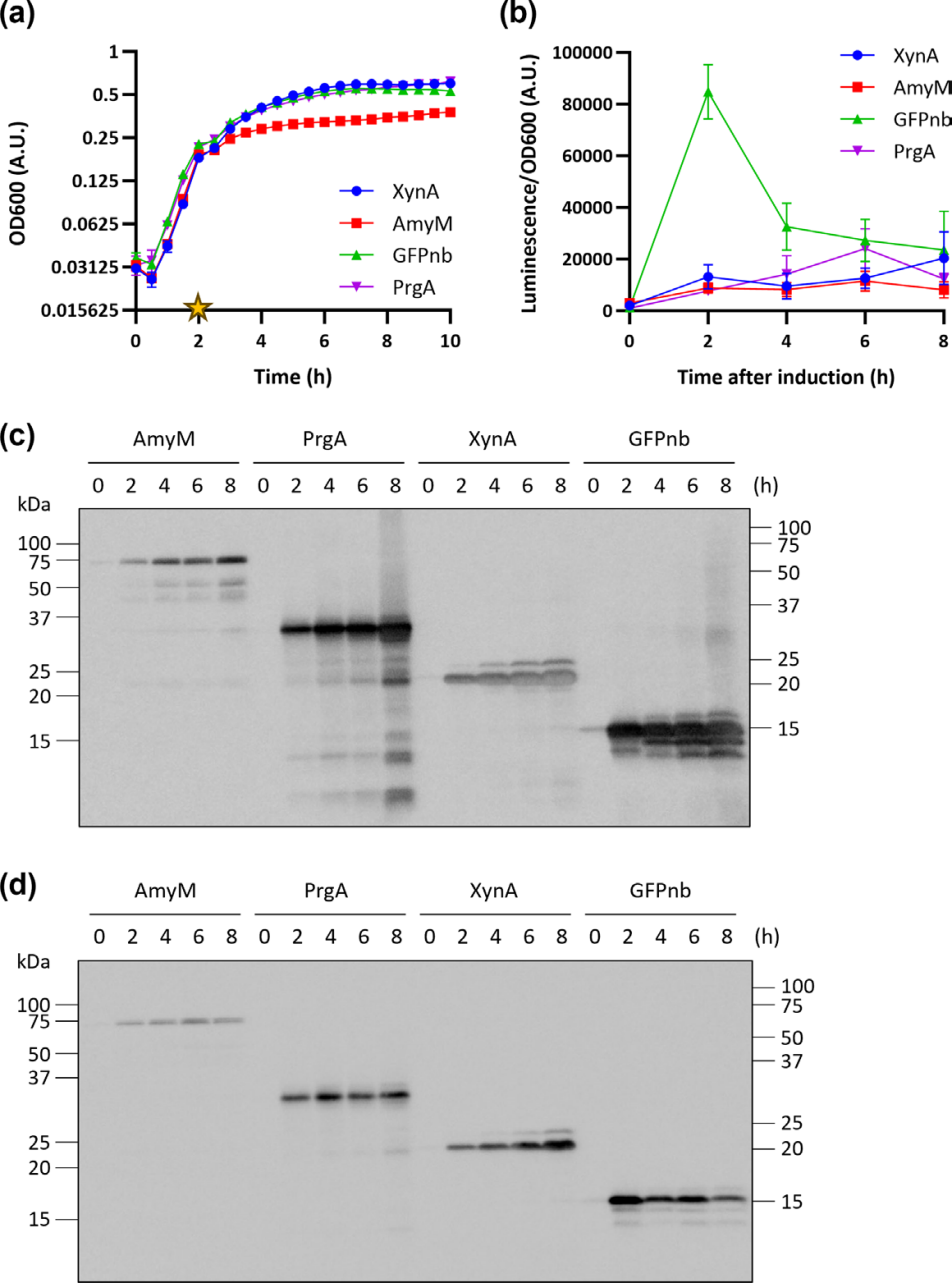
Detection of secreted HiBiT-tagged proteins using the split-luciferase assay and SDS-PAGE followed by (far-)Western blotting. BWB143 cells harbouring either pDEXH01 XynA-HiBiT, pDEXH02 AmyM-HiBiT, pDEXH03 GFPnb-HiBiT or pDEXH04 PrgA-HiBiT were induced at end log phase, and samples were taken at indicated hours after induction (h). **(a**) Growth curve. Yellow star depicts time point of induction. Error bars depict standard deviations of three biological replicates. (**b**) Culture samples were diluted 10-fold in LB and mixed with Nano-Glo reagent. After 10 min incubation time the luminescence signal was measured in a plate reader. Error bars depict standard deviation of three biological replicates. (**c**) Protein fractions of 200 µl supernatant equivalents at each sampling time point were separated by 12 % SDS-PAGE and transferred to a nitrocellulose membrane, which was incubated with α-HiBiT antibody, Lumi-Light Western blotting substrate and finally imaged for 0.1 s. Predicted molecular weights: AmyM-HiBiT 76.7 kDa, PrgA-HiBiT 34.3 kDa, XynA-HiBiT 22.0 kDa, GFPnb-HiBiT 15.3 kDa. (**d**) Protein fractions of 200 µl supernatant equivalents at each sampling time point were separated by 12 % SDS-PAGE and transferred to a nitrocellulose membrane, which was incubated with the Nano-Glo reagent and imaged for 30 s.

Subsequently, protein production dynamics of the four proteins were analysed during growth. BWB143 cells harbouring the different plasmids were cultivated in a 96-well plate to end log phase and protein production was induced by the addition of 1 % (w/v) xylose (Fig. 4a). Samples were collected at 0, 2, 4, 6, and 8 h post-induction, diluted in LB, and analysed with the luminescence assay (Figs 4b and S3a). The XynA-HiBiT and AmyM-HiBiT signals increased slowly over time, while the AmyM-HiBiT signal was weaker than the XynA-HiBiT signal. The PrgA-HiBiT signal started at similar levels to the AmyM-HiBiT, increased quickly, and overtook XynA-HiBiT at 4 h after induction, but decreased after that. GFPnb demonstrated the most efficient secretion, reaching a peak signal at 2 h post-induction. Its signal decreased at later time points. The decrease of signal from both PrgA-HiBiT and GFPnb-HiBiT at later time points potentially indicates a reduced availability of the tag or degradation of the construct.

To validate that the luminescence signals accurately reflect the levels and kinetics of protein secretion, we analysed supernatant fractions at the same time points by SDS-PAGE. We performed Western blotting using an anti-HiBiT antiserum (Fig. 4c), and far-Western blotting using the luminescence signal that originates from reconstituting NanoLuc on the nitrocellulose membrane (Fig. 4d). The quantified signal intensities are shown in Fig. S3b, c. Overall, the levels of protein detected with the two methods correspond to each other at the earliest time point, with higher levels of GFPnb being detected, whereas the levels of AmyM appeared markedly lower when compared to the other proteins. The XynA and AmyM levels increased over time, similar to the signal in the cultures. A marked difference is the level of GFPnb detected in the Western blot, which appeared to increase over time, whereas the far-Western blot levels followed the pattern of the in-plate luminescence assay. Furthermore, the peak signal of PrgA at 6 h after induction detected in the plate reader is not detectable by Western blotting. These discrepancies most likely arise from differences in the *in vivo* availability of the HiBiT tag for complementation with LgBiT in a (partly) folded state (Fig. 4b, d) as opposed to its presentation in an unfolded state on CBB-stained gel (Fig. 4c). Furthermore, since the bacterial cells are still present in the culture samples taken for the split-luciferase assay (Fig. 4b), secretion intermediates that are translocated but still attached to the cells could contribute to the luciferase signal. Thus, the luminescence values may not reflect overall protein levels, but are still indicative of successful secretion. Therefore, we suggest that direct in-culture luminescence determination be used to study proteins for screening purposes.

### Detection of improved AmyM-HiBiT secretion

**Fig. 5.**
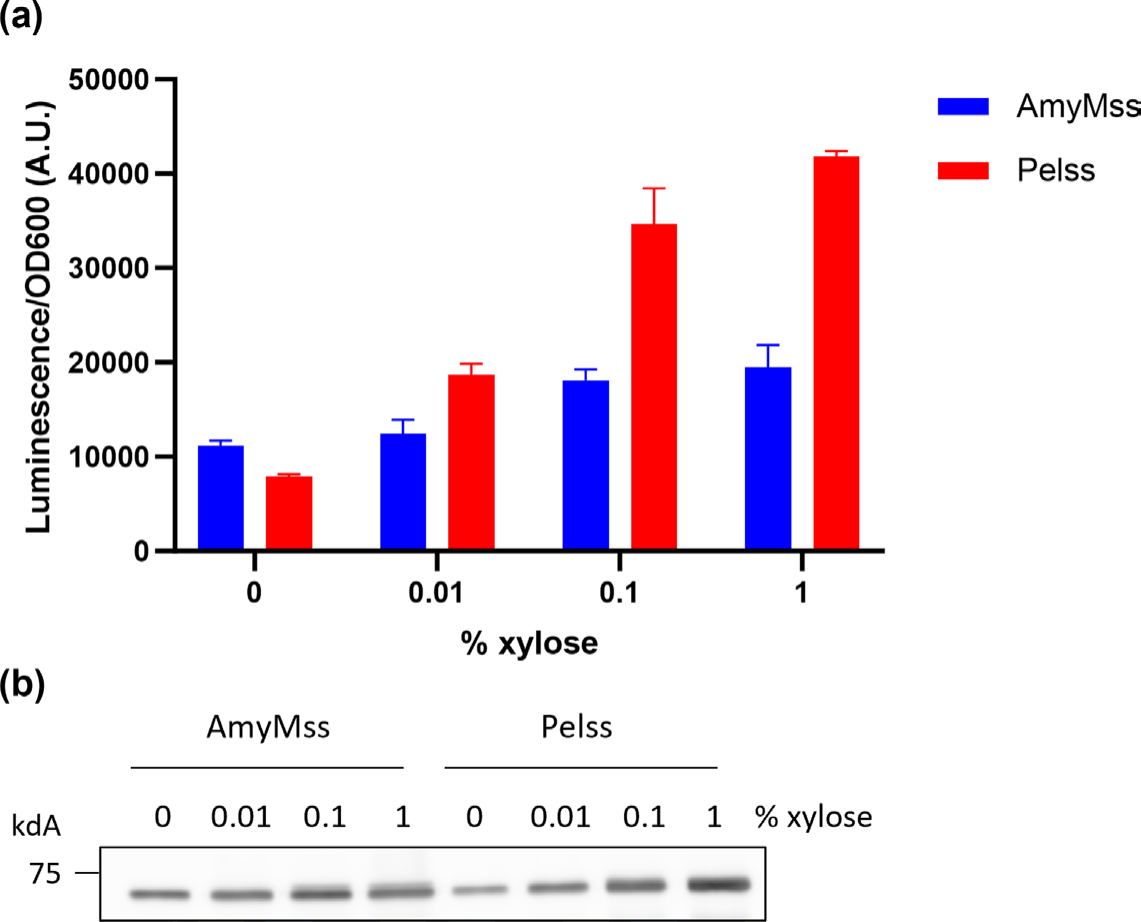
Luminescence signals of AmyMss-AmyM-HiBiT and Pelss-AmyM-HiBiT. BWB143 cells harbouring either pDEXH02 AmyMss-AmyM-HiBiT or pMKX09 Pelss-AmyM-HiBiT were induced with indicated concentrations of xylose at end log phase, and culture and supernatant samples were taken 4 h after induction. (**a**) The culture samples were diluted 10-fold in LB and mixed with Nano-Glo reagent. After 10 min incubation time the luminescence signal was measured in a plate reader. Error bars depict standard deviation of three biological replicates. (**b**) The supernatant samples were separated by 10 % SDS-PAGE and transferred to a nitrocellulose membrane, which was incubated with α-AmyM antibody and Lumi-Light Western blotting substrate, and finally imaged for 5 s. The equivalent of 0.15 OD_600_ units was loaded in each lane.

To demonstrate the utility of the split-luciferase system in monitoring protein secretion for the optimalization of a single protein, we chose to optimize AmyM secretion, which was selected based on its low detection levels in the preliminary assays. We substituted the native AmyM signal sequence (AmyMss) with the signal sequence of pectate lyase C (Pelss), a commonly employed signal sequence used for heterologous protein production in *B. subtilis* and other production hosts [9, 13,29,33]. We expressed the two AmyM constructs in the BWB143 strain using incremental inducer concentrations (Fig. S4a, b), and applied the medium-throughput 96-well plate assay, taking samples 4 h after induction. The Pel signal sequence clearly resulted in higher extracellular production levels with increasing inducer concentrations, as detected by the split-luciferase assay (Fig. 5a), which generally aligned with protein levels observed by Western blotting (Fig. 5b). These results showcase the applicability of the split-luciferase system for a swift comparative assessment of protein production using different conditions.

## Conclusion

We show here that the split-luciferase system can be used to set up a rapid and medium-throughput assay to monitor protein secretion by *B. subtilis*. Our data showed that the system has a high signal-to-noise ratio and is suitable to detect signals at different levels of protein production and representative of the protein levels detected with other methods. Furthermore, our comparison of two AmyM constructs underscores its applicability, rendering it a valuable addition to the protein detection toolbox.

## supplementary material

10.1099/mic.0.001460 Uncited Supplementary Material 1.

## Abbreviations

AmyM: amylase M
AmyMss: amylase M signal sequence
CBB: Coomassie brilliant blue
EV: empty vector
GFP: green fluorescent protein
GFPnb: nanobody targeting GFP
GRAS: generally recognized as safe
LB: lysogeny broth
OD_600_: optical density 600 nm
Pelss: pectate lyase C signal sequence
PMSF: phenylmethylsulfonyl fluoride
PrgA: protein glutaminase A
XynA: xylanase A
