# Supplementary material for: Development of a split-luciferase assay to establish optimal protein secretion conditions for protein production by Bacillus subtilis
Source: Microbiology (Reading). 2024 Jun 7;170(6):001460. doi: 10.1099/mic.0.001460 (PMC11261832; doi:10.1099/mic.0.001460)
Supplement: Uncited Supplementary Material 1. [file mic-170-01460-s001.pdf]

**Supplementary figures**

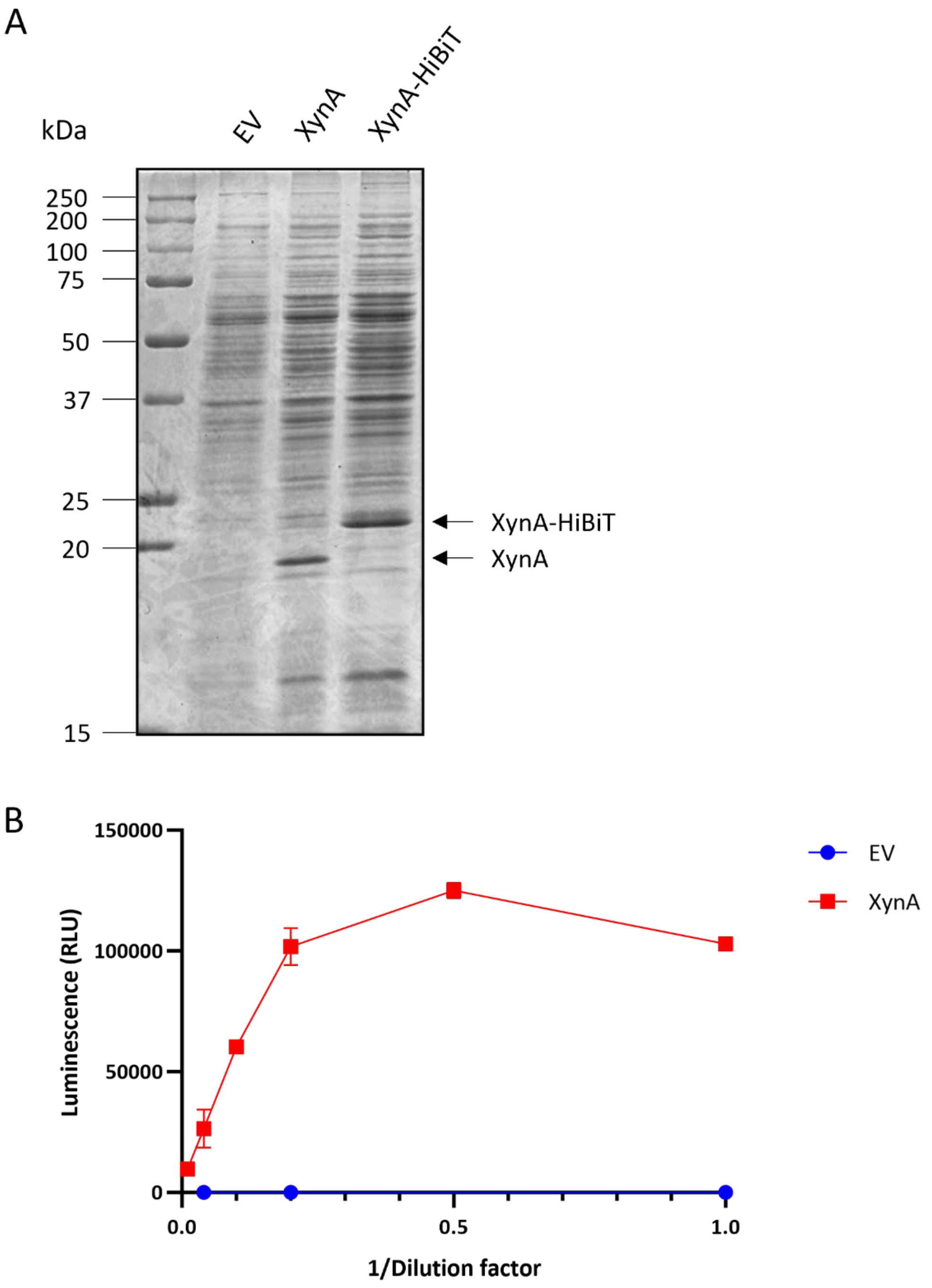


**Figure S1. Detection of secreted XynA-HiBiT by SDS-PAGE and the split-luciferase assay.** BWB143 cells harboring either pMKX01 EV, pMKX03 XynA or pDEXH01 XynA-HiBiT were grown from an equivalent of 0.05 $OD_{600}$ units to end log phase, and expression of the constructs was induced with 1% (w/v) xylose. **A**) 2 h after induction medium fractions were analyzed by SDS-PAGE and Coomassie Brilliant Blue staining. Equivalents of 0.5 $OD_{600}$ units were loaded. Arrows indicate predicted molecular weights of XynA and XynA-HiBiT (20.4 and 22.0 kDa, respectively). **B**) Samples from EV and XynA-HiBiT cultures were taken 1 h after induction and diluted in LB. The dilution range was incubated with Nano-Glo® reagent for 10 min and luminescence was measured in a plate reader. Error bars depict standard deviation of two technical replicates.

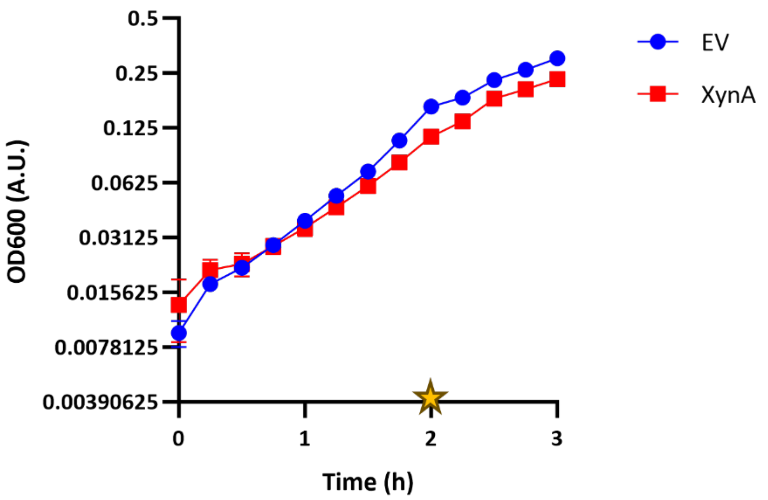


**Figure S2. Growth curve of XynA-HiBiT expression experiment.** BWB143 cells harboring either pMKX01 EV or pDEXH01 XynA-HiBiT were cultured in a plate reader and expression of the constructs was induced with 1% (w/v) xylose at end log phase (yellow star). Error bars depict standard deviations of three biological replicates.

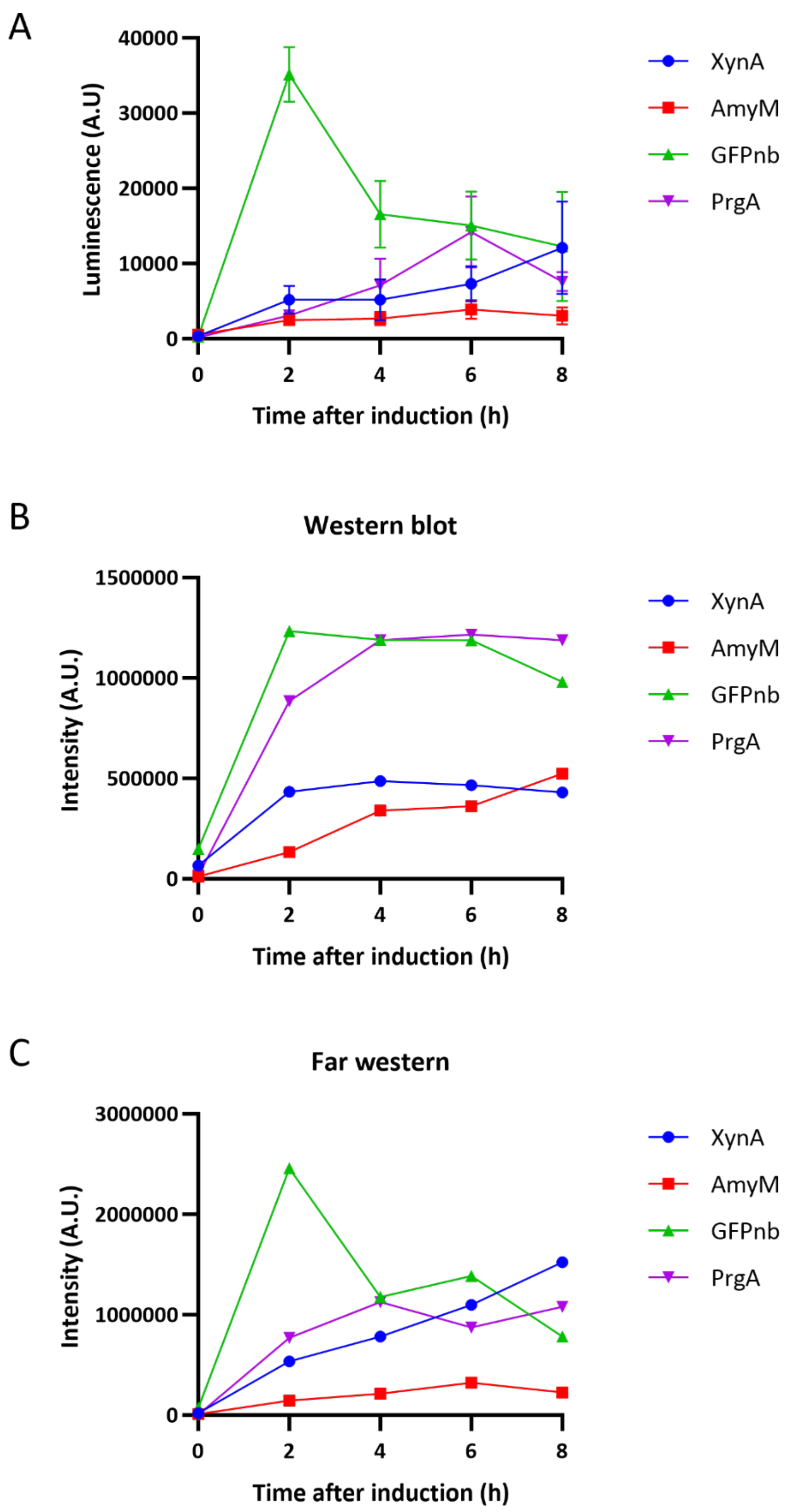


**Figure S3. Effect of induced expression of XynA-HiBiT, AmyM-HiBiT, GFPnb-HiBiT and PrgA-HiBiT.** BWB143 cells harboring either pDEXH01 XynA-HiBiT, pDEXH02 AmyM-HiBiT, pDEXH03 GFPnb-HiBiT or pDEXH04 PrgA-HiBiT were cultured in a plate reader and expression of the constructs was induced with 1% (w/v) xylose at end log phase. Culture samples were taken at indicated timepoints post induction. **A**) Culture samples were diluted 10-fold in LB and mixed with Nano-Glo® reagent. After 10 min incubation time the luminescence signal was measured in a plate reader. Error bars depict standard deviation of three biological replicates. **B-C**) Quantifications of (far-)western blots shown in Figure 3B and C.

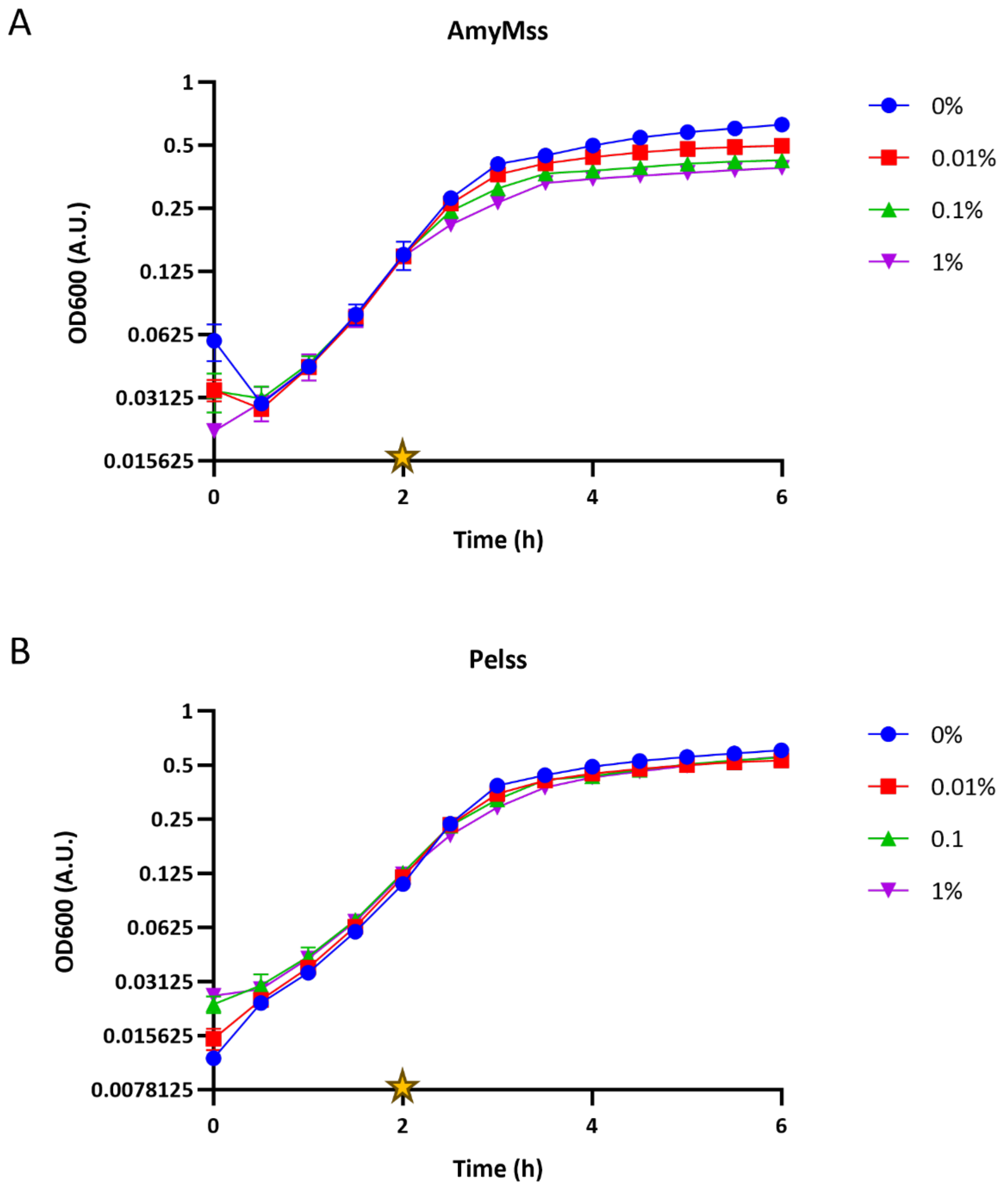


**Figure S4. Expression of AmyMss-AmyM-HiBiT and Pelss-AmyM-HiBiT.** BWB143 cells harboring either pDEXH02 AmyMss-AmyM-HiBiT (**A**) or pMKX09 Pelss-AmyM-HiBiT (**B**) were cultured in a plate reader and expression of the constructs was induced with indicated concentrations of xylose at end log phase (yellow star). Error bars depict standard deviations of three biological replicates.

## Supplementary methods

### Strain construction

Strain BWB143 (BSB1 trp+ Δ*aprE*, Δ*nprE*, Δ*spoIIE*) was created by sequentially transforming strain BSB1 with the appropriate PCR-amplified genomic regions from the BKE single deletion library [1] (**Table S1**). After transformation and validation of the knock-out, the erythromycin marker was removed by Cre-lox recombinase, encoded on the pDR244 plasmid as described by Koo *et al*. (2017) [1].

**Table S1: primers for strain construction**

| Mutant | Fw primer | Rv primer |
|---|---|---|
| *spoIIE* | EKP13 | BW_178 |
| *aprE* | BW_276 | BW_277 |
| *nprE* | BW_274 | BW_275 |

### Plasmid construction

All enzymes and buffers mentioned in this section were obtained from New England Biolabs (NEB) unless otherwise specified.

1. Xylose vectors (EV); Gibson cloning

Empty vector plasmid pMKX01 was constructed with a xylose-inducible promotor ($P_{xylR}$). A summary of the construction is shown in **Table S2**. In short, each fragment was obtained by PCR. Plasmid pCS74 was used as backbone, the $P_{xylR}$ cassette was constructed based on the chromosomal XylR, $P_{xylR}$ from pHJS105-GFP and terminator from pCS58. The fragments were combined by overlap extension PCR and Gibson assembly [2].

**Table S2: construction of empty vector pMKX01**

| Fragment | Primers | Template |
|---|---|---|
| Backbone | BW325 & BW326 | pCS74 EV [3] |
| XylR | BW319 & BW320 | *B. subtilis* 168 genome |
| $P_{xylR}$ (+RBS+ATG) | BW321 & BW322 | pHJS105-GFP [4] |
| XynA terminator | BW323 & BW324 | pCS58 [5] |

2. GFPnb and PrgA inserts; restriction-ligation cloning

The sequence of *prgA* from *Chryseobacterium proteolyticum* strain 9670 was partly codon-optimized by changing the codons that had a >2 fold different usage in *B. subtilis*. The sequence was extended with BamHI and XhoI restriction sites at the 5' and 3' ends of the sequence, respectively. The GFPnb sequence from [6] was used as template. The sequence was codon optimized for *Bacillus subtilis* by GeneArt Gene Synthesis (Thermo Fisher). The sequence was extended with BglII and PstI restriction sites at the 5' and 3' ends of the sequence, respectively. Both sequences were ordered via this service.

The synthetic DNA was firstly incorporated into a pJET vector (CloneJET PCR Cloning Kit, ThermoFisher). Next, *prgA* was cloned from the pJET vector into pJHS12 [7] using restriction enzymes mentioned in **Table S3**, *gfpnb* was cloned into the vector directly from the synthetic DNA.

**Table S3: construction of vectors encoding *prgA* and *gfpnb***

| Final product | Fragment | Enzymes | Template |
|---|---|---|---|
| pMKS04 PrgA | Backbone | BamHI & XhoI | pJHS12 [7] |
| pMKS04 PrgA | GOI | BamHI & PspXI | pJET PrgA |
| pMKS05 GFPnb | Backbone | BamHI & PstI | pJHS12 [7] |
| pMKS05 GFPnb | GOI | BglII & PstI | Synthetic GFPnb DNA |

3. Xylose vectors (inserts); Gibson cloning

The genes were inserted into pMKX01. The fragments were obtained by PCR (**Table S4**) and combined with Gibson assembly and overlap PCR (for combining SP and GFPnb/PrgA fragments).

**Table S4: construction of vectors encoding untagged genes**

| Final product | Fragment | Primers | Template |
|---|---|---|---|
| pMKX02 AmyM | Backbone | MK003 & MK004 | pMKX01 EV |
| pMKX02 AmyM | GOI | MK005 & MK006 | pCS73 AmyM [3] |
| pMKX03 XynA | Backbone | MK004 & MK007 | pMKX01 EV |
| pMKX03 XynA | GOI | MK008 & MK009 | pCS58 XynA [5] |
| pMKX04 GFPnb | Backbone | MK004 & MK012 | pMKX01 EV |
| pMKX04 GFPnb | SP | MK010 & MK011 | pMKS05 GFPnb |
| pMKX04 GFPnb | GOI | MK013 & MK019 | pMKS05 GFPnb |
| pMKX05 PrgA | Backbone | MK004 & MK012 | pMKX01 EV |
| pMKX05 PrgA | SP | MK010 & MK011 | pMKS05 GFPnb |
| pMKX05 PrgA | GOI | MK015 & MK019 | pMKS04 PrgA |

4. HiBiT constructs; Gibson cloning

The backbone for each plasmid was obtained by cutting pMKX03 XynA with AvrII and MluI. Plasmids inserts were obtained by PCR and inserts were inserted into the backbone by Gibson assembly.

An *E. coli* codon-optimized DNA sequence for the HiBiT tag was incorporated into the respective PCR primers (**Table S5**).

**Table S5: PCR fragments for HiBiT tagging**

| Final product | Fragment | Primers | Template |
|---|---|---|---|
| pDEXH01 XynA-HiBiT | XynA-HiBiT | DE001&DE002 | pMKX03 XynA |
| pDEXH01 XynA-HiBiT | HiBiT-terminator | DE003&DE004 | pMKX03 XynA |
| pDEXH02 AmyM-HiBiT | AmyMss-AmyM-HiBiT | DE001&DE005 | pMKX02 AmyM |
| pDEXH02 AmyM-HiBiT | HiBiT-terminator | DE003&DE004 | pMKX03 XynA |
| pDEXH03 GFPnb-HiBiT | GFPnb-HiBiT | DE001&DE006 | pMKX04 GFPnb |
| pDEXH03 GFPnb-HiBiT | HiBiT-terminator | DE003&DE004 | pMKX03 XynA |
| pDEXH04 PrgA-HiBiT | PrgA-HiBiT | DE001&DE007 | pMKX05 PrgA |
| pDEXH04 PrgA-HiBiT | HiBiT-terminator | DE003&DE004 | pMKX03 XynA |

5. AmyM constructs; Gibson cloning & restriction-ligation cloning

First, a His6 tag was added to AmyM in pMKX02 to obtain pNMX01 and then the signal sequence was replaced to obtain pNMX02. The backbone for pNMX01 and pNMX02 was obtained by cutting pMKX02 with AvrII & MluI. The inserts for pNMX01 and pNMX02 were obtained by PCR and inserted into the backbone via Gibson assembly (**Table S6**).

**Table S6: construction of AmyM-His6 derivatives**

| Final product | Fragment | Primers | Template |
|---|---|---|---|
| pNMX01 AmyMss-AmyM-His6 | AmyMss-AmyM | DE001 & MK006 | pMKX02 AmyM |
| pNMX01 AmyMss-AmyM-His6 | His6 | NM001 & DE004 | pMKS05 GFPnb |
| pNMX02 Pelss-AmyM-His6 | Pelss | NM006 & NM007 | *B. subtilis* 168 genome |
| pNMX02 Pelss-AmyM-His6 | AmyM-His6 | NM010 & DE004 | pNMX01 AmyM-His6 |

To obtain the plasmid encoding Pelss-AmyM-HiBiT, the -His6 tag from pNMX02 was replaced by the -HiBiT tag from pDEXH02 AmyM-HiBiT via restriction-ligation cloning using BamHI and MluI (**Table S7**).

**Table S7: construction of pMKX09 Pelss-AmyM-HiBiT**

| Fragment | Restriction enzymes | Template |
|---|---|---|
| HiBiT tag | BamHI & MluI | pDEXH02 AmyMss-AmyM-HiBiT |
| Backbone-Pelss-AmyM | BamHI & MluI | pNMX02 Pelss-AmyM-His6 |

Primer list

**Table S8: overview of primers used in this study**

| Primer name | Sequence |
|---|---|
| BW_178 | TATACTCTTTTATCGCCCGGC |
| BW_274 | CCTTTCCTGACTTTTGGAAC |
| BW_275 | ATGTTCAACTCGATTCGACG |
| BW_276 | GACTGAAACATCATCGGCAT |
| BW_277 | CAGTGGCCGAGCAGTATT |
| BW319 | GAGAGCAAAACCCCCCTTTGCTAAAGAAAAATAATTTGCACATGAAAA |
| BW320 | CACATTAGATATAATAAAGGGAAG |
| BW321 | GAATCTTCCCTTTATTATATCTAATGTGTTCATGAAAAACTAAAAAAAATATTGAAA |
| BW322 | CATCCTAGGAATCTCCTTTC |
| BW323 | TCTAGAAAGGAGATTCCTAGGATGTAACAGATCATCCTTAATCAGG |
| BW324 | CCTCCAGCAATTCCAAGG |
| BW325 | GGCCTTGGAATTGCTGGAGGGATCACGCGTTCTAGAGG |
| BW326 | GCAAAGGGGGGTTTTGCT |
| DE001 | CATAAAATGCATCTAGAAAGGAGATTC |
| DE002 | TTAAGAAATCTTCTTGAATAAGCGCCATCCGCTAACCGAGCCTGATCCCCACACTGTTACGTTAGAACTT |
| DE003 | GGATCAGGCTCGGTTAGCGGATGGCGCTTATTCAAGAAGATTTCTTAACAGATCATCCTTAATCAGGG |
| DE004 | GAGGTGAATTTCGACCTCTAGAA |
| DE005 | TTAAGAAATCTTCTTGAATAAGCGCCATCCGCTAACCGAGCCTGATCCGTTTTGCCACGTAACAGTAATG |
| DE006 | TTAAGAAATCTTCTTGAATAAGCGCCATCCGCTAACCGAGCCTGATCCTTTTGATGAAACTGTAACTTGT |
| DE007 | TTAAGAAATCTTCTTGAATAAGCGCCATCCGCTAACCGAGCCTGATCCAAATCCACAGCTGGAGACATCC |

| | |
|---|---|
| EKP13 | ATCAGCTGAAGAAACGAAGACG |
| MK003 | TAACATTACTGTTACGTGGCAAAACTAACAGATCATCCTTAATCAGG |
| MK004 | CATCCTAGGAATCTCCTTTC |
| MK005 | CATCTAGAAAGGAGATTCCTAGGATGAAAAAGAAAACGCTTTCTTTAT |
| MK006 | GTTTTGCCACGTAACAGTAAT |
| MK007 | GGAAGTTCTAACGTAACAGTGTGGTAACAGATCATCCTTAATCAG |
| MK008 | CATCTAGAAAGGAGATTCCTAGGATGTTTAAGTTTAAAAAGAATTTCTTAG |
| MK009 | CCACACTGTTACGTTAGAAC |
| MK010 | CATCTAGAAAGGAGATTCCTAGGATGAAAAAGATGTTGATGTTAGCT |
| MK011 | AGCCGAAGCTTCCCCTA |
| MK012 | AGCACCACCACCACCACCACTAACAGATCATCCTTAATCAGG |
| MK013 | ATGTAGGGGAAGCTTCGGCTGATATTGGCATTAATAGCGATC |
| MK015 | CATGTAGGGGAAGCTTCGGCTGATTCCAACGGGAATCAGG |
| MK019 | GTGGTGGTGGTGGTGGTGCTC |
| NM001 | TAACATTACTGTTACGTGGCAAAACCTGCAGCAGGTCAGCG |
| NM006 | GCATCTAGAAAGGAGATTCCTAGGATGAAAAAAGTGATGTTAGCTAC |
| NM007 | TGCGTTCGCGCCAGCT |
| NM010 | CCAGCTGGCGCGAACGCAAGCAGTTCCGCAAGCGT |

## Supplementary references


[1] Koo BM, Kritikos G, Farelli JD, Todor H, Tong K, Kimsey H, et al. Construction and Analysis of Two Genome-Scale Deletion Libraries for Bacillus subtilis. Cell Syst 2017;4:291-305.e7. https://doi.org/10.1016/j.cels.2016.12.013.

[2] Gibson DG, Young L, Chuang RY, Venter JC, Hutchison CA, Smith HO. Enzymatic assembly of DNA molecules up to several hundred kilobases. Nat Methods 2009;6:343–5. https://doi.org/10.1038/nmeth.1318.

[3] Henriques G, McGovern S, Neef J, Antelo-Varela M, Götz F, Otto A, et al. SppI Forms a Membrane Protein Complex with SppA and Inhibits Its Protease Activity in Bacillus subtilis. MSphere 2020;5. https://doi.org/10.1128/msphere.00724-20.

[4] Jahn N, Brantl S, Strahl H. Against the mainstream: The membrane-associated type I toxin BsrG from Bacillus subtilis interferes with cell envelope biosynthesis without increasing membrane permeability. Mol Microbiol 2015;98:651–66. https://doi.org/10.1111/mmi.13146.

[5] Wang B, van der Kloet F, Hamoen LW. Induction of the CtsR regulon improves Xylanase production in Bacillus subtilis. Microb Cell Fact 2023;22. https://doi.org/10.1186/s12934-023-02239-3.

[6] van den Berg van Saparoea HB, Houben D, de Jonge MI, Jong WSP, Luirink J. Display of recombinant proteins on bacterial outer membrane vesicles by using protein ligation. Appl Environ Microbiol 2018;84. https://doi.org/10.1128/AEM.02567-17.

[7] Heinrich J, Drewniok C, Neugebauer E, Kellner H, Wiegert T. The YoaW signal peptide directs efficient secretion of different heterologous proteins fused to a StrepII-SUMO tag in Bacillus subtilis. Microb Cell Fact 2019;18:1–14. https://doi.org/10.1186/s12934-019-1078-0.
